# Does Simplicity Compromise Accuracy in ACS Risk Prediction? A Retrospective Analysis of the TIMI and GRACE Risk Scores

**DOI:** 10.1371/journal.pone.0007947

**Published:** 2009-11-23

**Authors:** Krishna G. Aragam, Umesh U. Tamhane, Eva Kline-Rogers, Jin Li, Keith A. A. Fox, Shaun G. Goodman, Kim A. Eagle, Hitinder S. Gurm

**Affiliations:** 1 Division of Cardiovascular Medicine, University of Michigan, Ann Arbor, Michigan, United States of America; 2 Cardiovascular Research, Division of Medical and Radiological Sciences, The University of Edinburgh, Edinburgh, Scotland; 3 Canadian Heart Research Centre and Terrence Donnelly Heart Centre, Division of Cardiology, St Michael's Hospital, University of Toronto, Toronto, Ontario, Canada; Lerner Research Institute, Cleveland Clinic, United States of America

## Abstract

**Background:**

The Thrombolysis in Myocardial Infarction (TIMI) risk scores for Unstable Angina/Non-ST–elevation myocardial infarction (UA/NSTEMI) and ST-elevation myocardial infarction (STEMI) and the Global Registry of Acute Coronary Events (GRACE) risk scores for in-hospital and 6-month mortality are established tools for assessing risk in Acute Coronary Syndrome (ACS) patients. The objective of our study was to compare the discriminative abilities of the TIMI and GRACE risk scores in a broad-spectrum, unselected ACS population and to assess the relative contributions of model simplicity and model composition to any observed differences between the two scoring systems.

**Methodology/Principal Findings:**

ACS patients admitted to the University of Michigan between 1999 and 2005 were divided into UA/NSTEMI (n = 2753) and STEMI (n = 698) subpopulations. The predictive abilities of the TIMI and GRACE scores for in-hospital and 6-month mortality were assessed by calibration and discrimination. There were 137 in-hospital deaths (4%), and among the survivors, 234 (7.4%) died by 6 months post-discharge. In the UA/NSTEMI population, the GRACE risk scores demonstrated better discrimination than the TIMI UA/NSTEMI score for in-hospital (C = 0.85, 95% CI: 0.81–0.89, versus 0.54, 95% CI: 0.48–0.60; p<0.01) and 6-month (C = 0.79, 95% CI: 0.76–0.83, versus 0.56, 95% CI: 0.52–0.60; p<0.01) mortality. Among STEMI patients, the GRACE and TIMI STEMI scores demonstrated comparably excellent discrimination for in-hospital (C = 0.84, 95% CI: 0.78–0.90 versus 0.83, 95% CI: 0.78–0.89; p = 0.83) and 6-month (C = 0.72, 95% CI: 0.63–0.81, versus 0.71, 95% CI: 0.64–0.79; p = 0.79) mortality. An analysis of refitted multivariate models demonstrated a marked improvement in the discriminative power of the TIMI UA/NSTEMI model with the incorporation of heart failure and hemodynamic variables. Study limitations included unaccounted for confounders inherent to observational, single institution studies with moderate sample sizes.

**Conclusions/Significance:**

The GRACE scores provided superior discrimination as compared with the TIMI UA/NSTEMI score in predicting in-hospital and 6-month mortality in UA/NSTEMI patients, although the GRACE and TIMI STEMI scores performed equally well in STEMI patients. The observed discriminative deficit of the TIMI UA/NSTEMI score likely results from the omission of key risk factors rather than from the relative simplicity of the scoring system.

## Introduction

Risk stratification is integral to the management of patients presenting with Acute Coronary Syndromes (ACS). Current AHA/ACC guidelines promote the use of the Thrombolysis in Myocardial Infarction (TIMI) and Global Registry of Acute Coronary Events (GRACE) risk scores to evaluate the in-hospital and post-discharge risk of ACS patients [1]. Both of these scoring systems have been shown to predict the response of ACS patients to various treatment modalities, and may therefore significantly influence therapeutic decision-making [2], [3], [4]. The TIMI risk scores for Unstable Angina/Non ST-Elevation Myocardial Infarction (UA/NSTEMI) and for ST-Elevation Myocardial Infarction (STEMI) patients are simple, integer-based scores derived from selected clinical-trial cohorts [2], [5]. Though slightly more complex, the GRACE risk scores for in-hospital and 6-month mortality are derived from a more representative community-based registry [6], [7]. Recent studies have suggested the superiority of the GRACE risk scores as compared to the TIMI UA/NSTEMI score in UA and/or NSTEMI patients [8], [9], [10]. Comparisons of the TIMI and GRACE scores in STEMI patients remain unexplored.

This study aimed to evaluate the prognostic abilities of the TIMI and GRACE risk scores over a broad-spectrum of community-derived ACS patients (UA/NSTEMI and STEMI) admitted to a tertiary care center. Moreover, we sought to investigate the relative contributions of model simplicity and model composition to any observed prognostic differences between the TIMI and GRACE risk scores.

## Methods

### Study population

The study sample consisted of 3451 consecutive patients admitted to the University of Michigan between January 1999 and December 2005 with a discharge diagnosis of ACS. ACS was defined as presentation with symptoms of ischemia along with qualifying electrocardiographic changes, positive cardiac enzymes, new documentation of coronary artery disease (CAD) or prior existence of CAD.

The protocol was approved by the institutional review board at the University of Michigan. Informed consent was obtained for all patients enrolled after January 1, 2005 following enactment of the HIPAA Privacy Rule. Patient consent was either written or verbal; as per the institutional review board, verbal consent was obtained from subjects who did not return a written consent and/or did not opt out of the registry. Data were collected by trained personnel (physicians/nurses/medical residents) from review of hospital medical records using a standardized six-page case report form. Demographic characteristics, medical history, presenting symptoms, duration of pre-hospital delay, biochemical and electrocardiography findings, treatment practices and a variety of hospital outcome data were obtained. Standardized definitions of all patient-related variables and clinical diagnoses were used. All cases of acute coronary syndromes were assigned to one of the following categories: ST-elevation myocardial infarction, non-ST elevation myocardial infarction, or unstable angina. The outcomes of the study were all-cause in-hospital and six-month mortality, obtained by six-month telephone follow-up survey and the Social Security Death Index. In-hospital mortality data were available for 3451 patients and six-month mortality data for 3170 patients. For analysis, the ACS cohort was divided into UA/NSTEMI and STEMI subpopulations.

### Risk score calculation

All risk scores were calculated from available clinical data on patient presentation.

#### TIMI UA/NSTEMI score

The TIMI UA/NSTEMI risk score (range 0–7) was derived from a cohort of the TIMI 11B clinical trial for a composite endpoint of mortality, recurrent MI, and repeat revascularization at 14 days, and consists of seven equally-weighted, dichotomous variables (Table 1) [2]. In our database, history of angina was the closest substitute for ≥2 anginal episodes within the past 24 h; a sensitivity analysis yielded no change in the overall c-statistic, indicating adequate variable substitution.

**Table 1:** TIMI UA/NSTEMI risk score.

TIMI UA/NSTEMI RISK SCORE	
**1) Age ≥65**	1 point
**2) ≥3 risk factors for CAD**	1 point
**3) Use of ASA (last 7 days)**	1 point
**4) Known CAD (prior stenosis ≥50%)**	1 point
**5) >1 episode rest angina in <24 h**	1 point
**6) ST-segment deviation**	1 point
**7) Elevated cardiac markers**	1 point

#### TIMI STEMI score

The TIMI STEMI risk score (range 0–14) was derived from the Intravenous nPA for Infarcting Myocardium Early II (IN-TIME II) trial study population to predict all-cause mortality at 30 days and includes eight variables of differing weights (Table 2) [5].

**Table 2:** TIMI STEMI risk score.

TIMI STEMI RISK SCORE	
1) Age 65–74/>75	2/3 points
2) Systolic Blood Pressure <100	3 points
3) Heart Rate >100	2 points
4) Killip class II-IV	2 points
5) Anterior STE or LBBB	1 point
6) Diabetes, h/o HTN, or h/o angina	1 point
7) Weight <67 kg	1 point
8) Time to treatment >4 hours	1 point

#### GRACE in-hospital and 6-month scores

The GRACE in-hospital risk score (range 0–372) and the GRACE 6-month risk score (range 0–263) were derived from the GRACE registry for the endpoint of all-cause mortality and consist of eight and nine variables, respectively (Table 3), with published nomograms outlining the conversion of each variable to its corresponding point value [6], [7].

**Table 3:** GRACE in-hospital and 6-month risk score variables.

GRACE Risk Score Variables	
In-hospital risk score	6-month risk score
1) Age	1) Age
2) Heart Rate	2) H/o Congestive Heart Failure
3) Systolic Blood Pressure	3) H/o Myocardial Infarction
4) Serum Creatinine level	4) Heart Rate
5) Killip class	5) Systolic Blood Pressure
6) Cardiac arrest at admission	6) ST-segment depression
7) Elevated cardiac markers	7) Serum Creatinine
8) ST-segment deviation	8) Elevated cardiac markers
	9) No In-hospital PCI

The TIMI UA/NSTEMI risk score was determined for all UA/NSTEMI patients and compared against the patients' corresponding GRACE in-hospital and 6-month risk scores to determine the overall performance – discrimination and calibration – of each risk score for predicting in-hospital and 6-month mortality. Similarly, the TIMI STEMI, GRACE in-hospital and GRACE 6-month risk scores were assessed in STEMI patients. Comparisons between the GRACE and TIMI scores were performed using calculated risk scores and refitted multivariate logistic models. For the latter approach, original risk score variables were regressed against a given endpoint (in-hospital or 6-month mortality) with adjusted beta-coefficients for each variable to optimize model discrimination within the study population. Established prognostic variables were then added to the TIMI UA/NSTEMI multivariate model to evaluate their incremental benefit to model discrimination [11].

### Clinical end point

The primary end points of the study were all cause in-hospital and 6-month mortality.

### Statistical analysis

Discrimination indicates the ability of a model to distinguish between two outcomes and is reported by the c-statistic, equal to the area under the receiver-operating characteristic (ROC) curve. C-statistics were calculated to compare the discriminative capacities of different risk scores for in-hospital and 6-month mortality [12]. The areas under correlated ROC curves were compared by a nonparametric approach [13].

The Bayesian Information Criterion (BIC) and the Akaike Information Criterion (AIC) are variants of the likelihood ratio that adjust for the number of variables in a model [14]. These statistics were reported to further assess the incremental contribution of particular risk factors to the TIMI UA/NSTEMI risk model.

Calibration assesses the degree of correspondence between predicted and observed outcomes and can be measured by the Hosmer-Lemeshow (H-L) statistic for goodness-of-fit. The H-L statistic was obtained for each risk score and multivariate model along with its associated p-value; p>0.05 denoted insignificant difference from the line of perfect agreement and thus good model fit for the indicated population [15].

Data were analyzed using SAS version 8.2 (Cary, NC). The authors had full access to all data in the study and take responsibility for the integrity of the data and the accuracy of the analysis.

## Results

### Demographics

Patient demographics for the UA/NSTEMI and STEMI populations within the total ACS cohort are presented in Table 4. UA/NSTEMI patients comprised 75% of the ACS cohort. Fifty percent of all UA/NSTEMI patients were elderly (age ≥65 years) compared to 38% of STEMI patients. The age and gender demographics roughly matched those of the four risk score derivation cohorts; co-morbidities such as hypertension and hyperlipidemia were of comparable prevalence to the GRACE derivation cohorts, but more prevalent than in the TIMI derivation cohorts (Table S1). For the UA/NSTEMI subpopulation, in-hospital data were available in 2753 patients and six-month follow-up data in 2545 patients. For the STEMI cohort, in-hospital data were available in 698 patients and six-month follow-up data in 625 patients. The in-hospital mortality rate was 4% (n = 137) and the six-month mortality rate was 7.4% (n = 234).

**Table 4:** Baseline characteristics of patients in UA/NSTEMI and STEMI subpopulations.

Clinical Characteristics	UA/NSTEMI n = 2753	STEMI n = 698
Age ≥65 yrs, n (%)	1378 (50.1)	267 (38.3)
Female, n (%)	1013 (36.8)	216 (31.0)
**Medical History, n (%)**		
Myocardial infarction (MI)	1262 (45.8)	166 (23.8)
Congestive heart failure (CHF)	583 (21.2)	67 (9.6)
Hypertension	1989 (72.3)	404 (57.9)
Hyperlipidemia	1813 (65.9)	337 (48.3)
Coronary artery bypass graft	684 (24.9)	58 (8.3)
Peripheral arterial disease	408 (14.8)	62 (8.9)
Atrial fibrillation	302 (11.0)	34 (4.9)
Transient ischemic attack/Stroke	320 (11.6)	54 (7.7)
Current smoker	569 (20.7)	243 (34.8)
Diabetes	896 (32.6)	160 (22.9)
Renal insufficiency	427 (15.5)	50 (7.2)
**Presentation**		
Cardiac arrest, n (%)	40 (1.5)	38 (5.5)
Killip class >II, n (%)	430 (15.6)	112 (16.1)
Blood pressure systolic, mm Hg(mean±SD)	141.8±30.0	134.7±31.8
Blood pressure diastolic, mm Hg (mean±SD)	77.9±20.3	78.9±19.2
Heart Rate, beats/min (mean±SD)	79.6±21.4	81.2±24.4
ST segment depression, n (%)	409 (14.9)	252 (36.1)
Initial Creatinine, mg/dL(mean±SD)	1.3±1.1	1.2±0.9
**In-Hospital events, n (%)**		
Congestive heart failure/Pulmonary edema	224 (8.1)	100 (14.3)
Cardiogenic shock	104 (3.8)	73 (10.5)
Cardiac arrest/Ventricular fibrillation	87 (3.2)	71 (10.2)
Sustained ventricular tachycardia	43 (1.6)	15 (2.2)
Atrial fibrillation/flutter	171 (6.2)	63 (9.0)
Myocardial infarction	131 (4.8)	46 (6.6)
Stroke	18 (0.7)	11 (1.6)
Major bleeding/Hemorrhagic stroke	148 (5.4)	57 (8.2)
**In hospital therapies, n (%)**		
Percutaneous coronary intervention (PCI)	1049 (38.1)	494 (70.8)
Aspirin	2661 (96.7)	680 (97.4)
Clopidogrel/Ticlopidine	1071 (38.9)	352 (50.4)
Glycoprotein IIb/IIIa receptor agonists	628 (22.8)	311 (44.6)
Angiotensin receptor blockers/Angiotensin converting enzyme inhibitors	1894 (68.8)	577 (82.7)
Beta blockers (IV or oral)	2491 (90.5)	655 (93.8)
Statins	1982 (72.0)	534 (76.5)
**Discharge medications, n (%)**		
Aspirin	2455 (89.2)	623 (89.3)
Clopidogrel/Ticlopidine	1061 (38.5)	340 (48.7)
Angiotensin receptor blockers/Angiotensin converting enzyme inhibitors	1705 (61.9)	526 (75.4)
Beta blockers	2254 (81.9)	591 (84.7)
Statins	1973 (71.7)	528 (75.6)
In-hospital mortality rate, % (n)	3.41 (94)	6.16 (43)
Six-month mortality rate, % (n)	7.54 (192)	6.72 (42)

### Risk score comparisons

Calibration plots of observed versus predicted mortality (Figure S1 and Figure S2) and H-L statistics (Table 5) for the TIMI and GRACE risk scores suggested adequate calibration of all four risk scores for the ACS subpopulations in which they were employed.

**Table 5:** Hosmer-Lemeshow calibration statistics for the TIMI and GRACE risk scores.

Risk scores	P-values	Hosmer-Lemeshow Statistics
TIMI NSTEMI (in-hospital)	0.42	8.14
TIMI NSTEMI (6-month)	0.78	4.83
TIMI STEMI (in-hospital)	0.59	6.55
TIMI STEMI (6-month)	0.83	4.29
GRACE-in hospital (NSTEMI)	0.67	5.77
GRACE-in hospital (STEMI)	0.41	8.23
GRACE-6-month (NSTEMI)	0.68	5.72
GRACE-6-month (STEMI)	0.68	5.70

#### UA/NSTEMI

**Figure 1:**
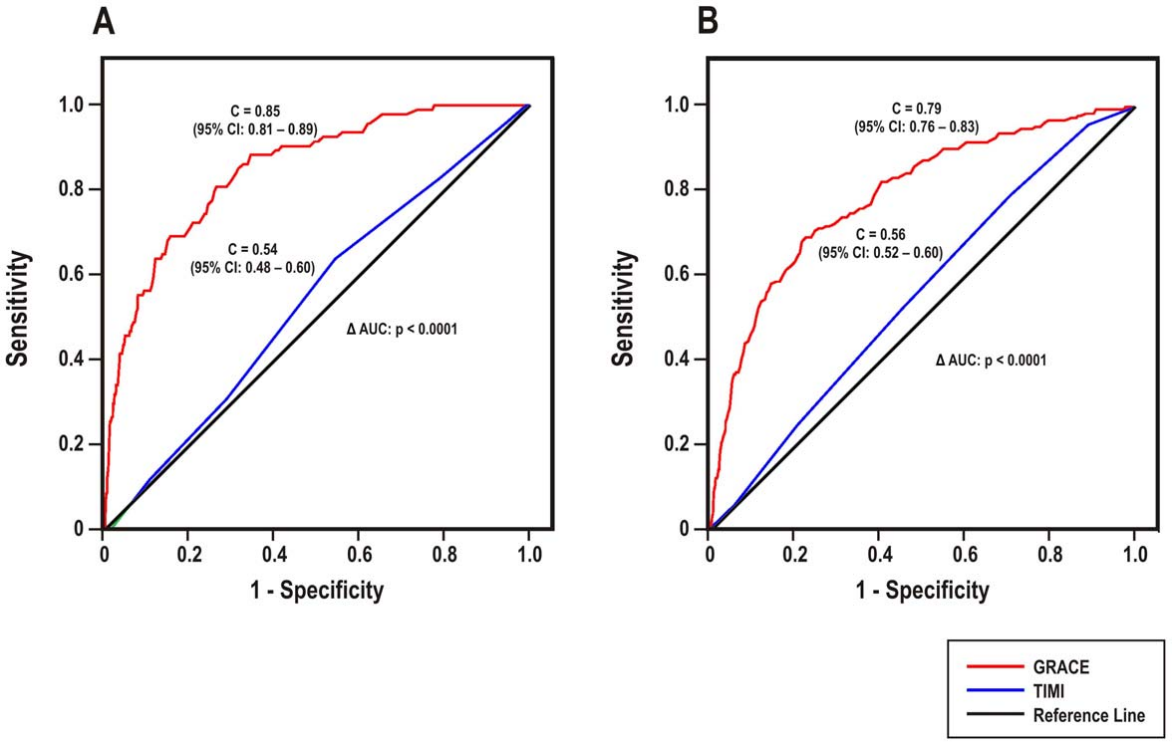
Comparison of TIMI UA/NSTEMI and GRACE risk scores in UA/NSTEMI patients. Receiver operating characteristic curves of (A) the TIMI UA/NSTEMI and GRACE in-hospital risk scores for predicting in-hospital mortality, and (B) the TIMI UA/NSTEMI and GRACE 6-month risk scores for predicting 6-month mortality in patients surviving to hospital discharge.

The ROC curves of the appropriate TIMI and GRACE risk scores for UA/NSTEMI patients are displayed in Figure 1. For in-hospital mortality (Figure 1A), the TIMI UA/NSTEMI score yielded a c-statistic of 0.54 (95% CI: 0.48–0.60) while the c-statistic for the GRACE in-hospital score was 0.85 (95% CI: 0.81–0.89). For 6-month mortality (Figure 1B), the c-statistics were 0.56 (95% CI: 0.52–0.60) for the TIMI UA/NSTEMI score and 0.79 (95% CI: 0.76–0.83) for the GRACE 6-month score. For each time-point, the GRACE risk score displayed significantly better discrimination than the TIMI score (p<0.0001, both comparisons).

**Figure 2:**
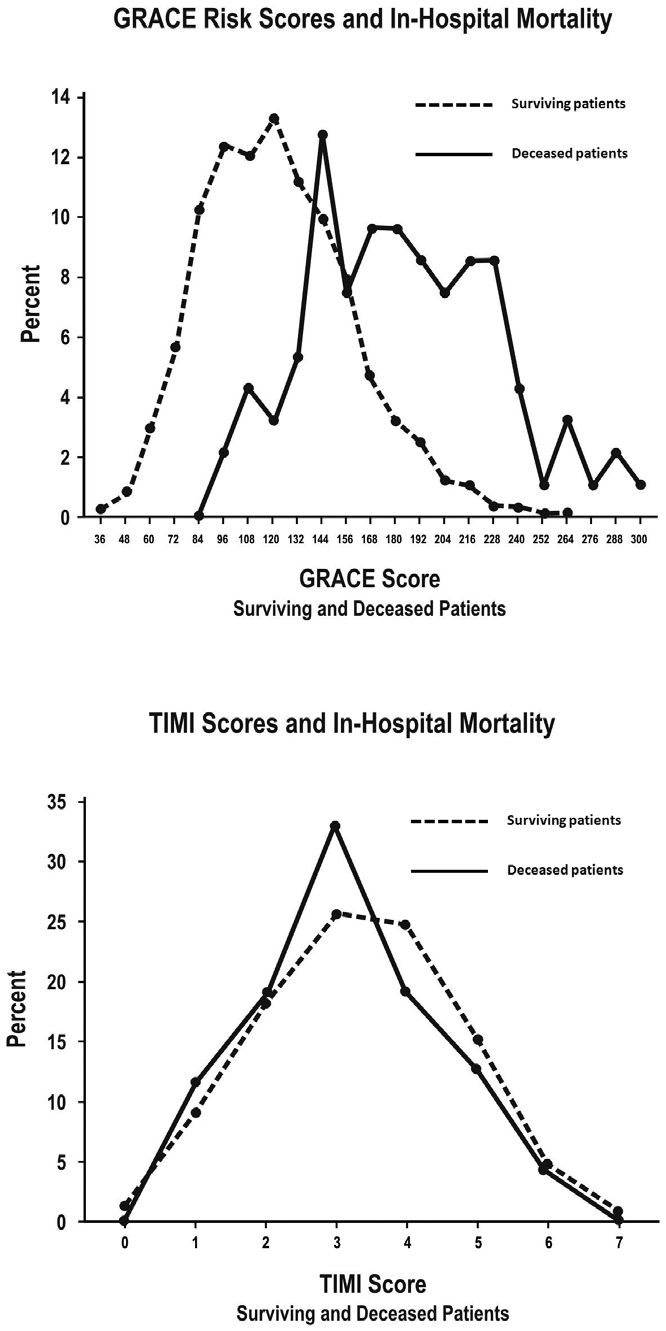
Risk score distributions of UA/NSTEMI patients for in-hospital mortality. (A) GRACE in-hospital and (B) TIMI UA/NSTEMI risk score distributions for surviving versus deceased UA/NSTEMI patients for in-hospital mortality.

**Figure 3:**
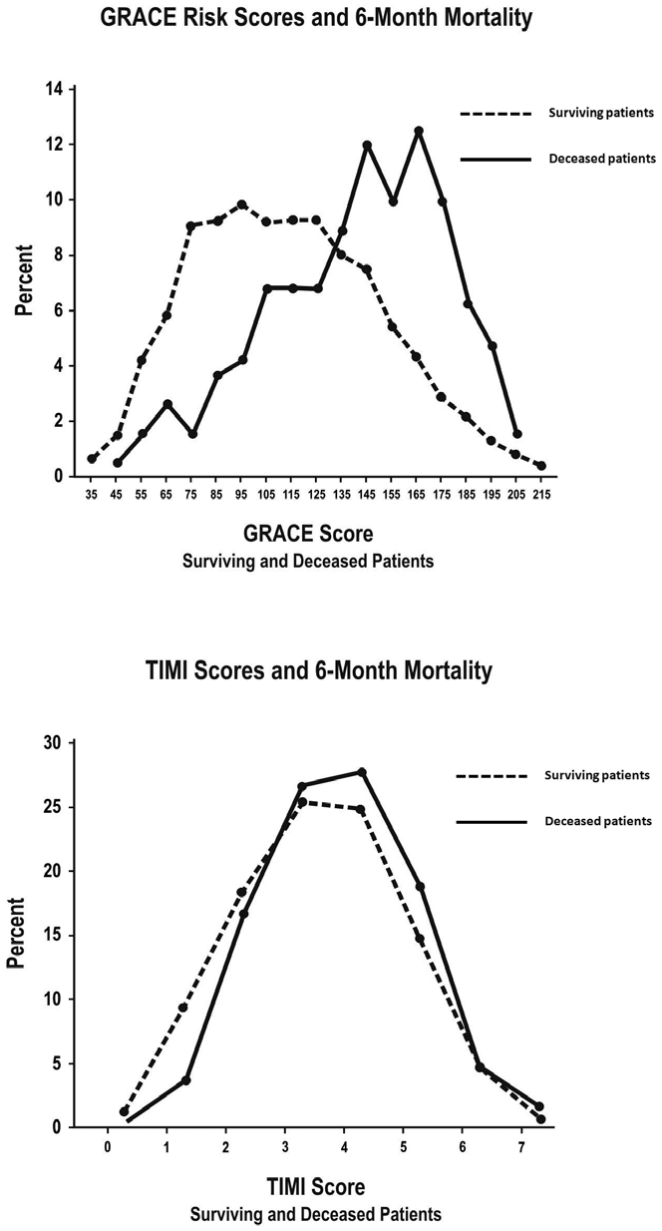
Risk score distributions of UA/NSTEMI patients for 6-month mortality. (A) GRACE 6-month and (B) TIMI UA/NSTEMI risk score distributions for surviving versus deceased UA/NSTEMI patients for 6-month mortality.

Comparisons of GRACE and TIMI score distributions between deceased and surviving UA/NSTEMI patients (Figure 2 and Figure 3) confirmed the above disparities in discrimination. At both the in-hospital (Figure 2) and 6-month (Figure 3) timepoints, there was a clearer differentiation in GRACE score distributions than in TIMI UA/NSTEMI score distributions between deceased and surviving patients.

#### STEMI

**Figure 4:**
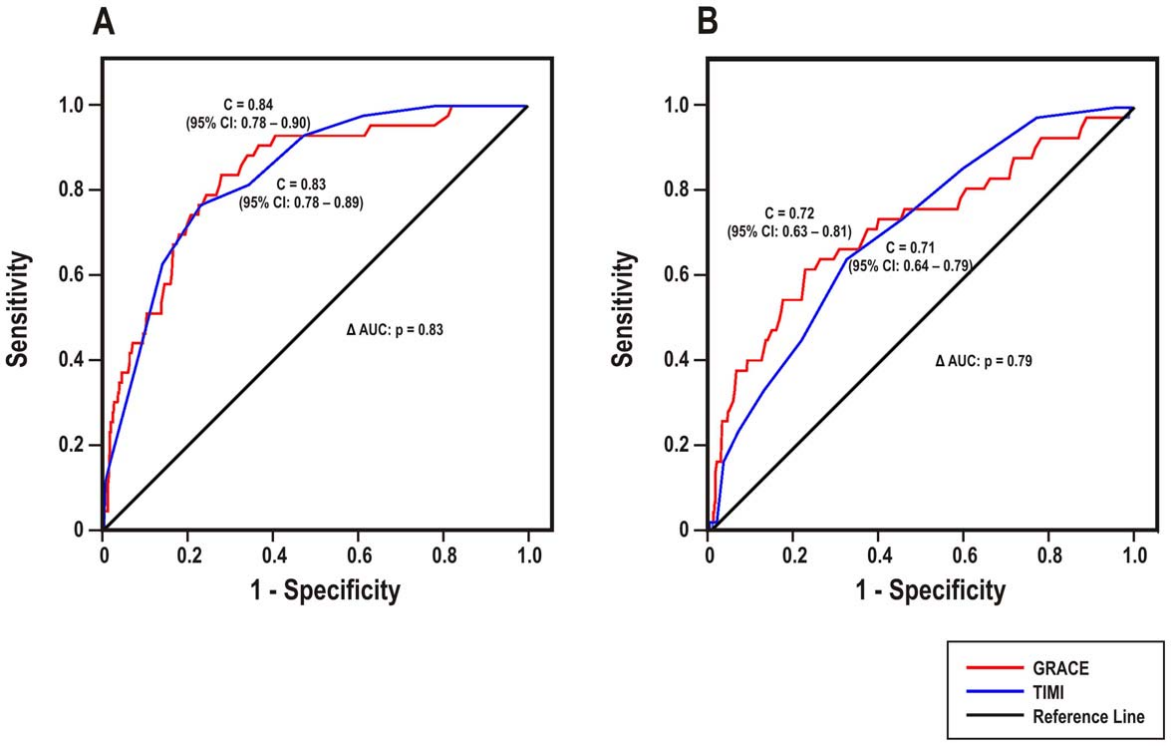
Comparison of TIMI STEMI and GRACE risk scores in STEMI patients. Receiver operating characteristic curves of (A) the TIMI STEMI and GRACE in-hospital risk scores for predicting in-hospital mortality, and (B) the TIMI STEMI and GRACE 6-month risk scores for predicting 6-month mortality in patients surviving to hospital discharge.

In STEMI patients (Figure 4), the TIMI STEMI and GRACE in-hospital scores yielded c-statistics of 0.83 (95% CI: 0.78–0.89) and 0.84 (95% CI: 0.78–0.90), respectively, for in-hospital mortality (Figure 3A). At 6-months (Figure 3B), the TIMI STEMI score produced a c-statistic of 0.71 (95% CI: 0.64–0.79) and the GRACE 6-month score yielded a c-statistic of 0.72 (95% CI: 0.63–0.81). There was no statistical difference in discrimination between the appropriate TIMI and GRACE risk scores for in-hospital and 6-month mortality (p = 0.83 and 0.79, respectively).

Consistent with these findings, there was comparable differentiation in the GRACE and TIMI score distribution curves between deceased and surviving STEMI patients at the in-hospital and 6-month timepoints (data not shown).

### Full logistic models: TIMI UA/NSTEMI analysis

To investigate the observed differences in discrimination between the TIMI and GRACE risk scores in UA/NSTEMI patients, full multivariate models of the TIMI UA/NSTEMI and GRACE in-hospital and 6-month scores were refitted to the UA/NSTEMI study population; calibration was assessed, and relative discriminative capacities were then compared for in-hospital and 6-month mortality. For in-hospital mortality, both models were well-calibrated to the study population (Figure S3A and S3C, Table 6). The c-statistic of the fitted TIMI UA/NSTEMI model (0.70, 95% CI: 0.65–0.75; Figure S4A) was significantly greater than that for the TIMI UA/NSTEMI risk score (C = 0.54, Figure 1A). The c-statistic for the GRACE in-hospital multivariate model also improved (0.90, 95% CI: 0.87–0.93; Figure S4A), and was significantly greater than its TIMI UA/NSTEMI counterpart (p<0.0001). Although the TIMI UA/NSTEMI multivariate model did not calibrate well (Figure S3B, Table 6), it too displayed a marked improvement in discrimination (C = 0.70, 95% CI: 0.66–0.74; Figure S4B) as compared to the corresponding risk score (C = 0.56, Figure 1B); however, it was significantly inferior to the GRACE 6-month multivariate model (0.84, 95% CI: 0.81–0.87; p<0.0001).

**Table 6:** Hosmer-Lemeshow calibration statistics for the TIMI UA/NSTEMI and GRACE refitted multivariate models.

Risk scores	P-values	Hosmer-Lemeshow Statistics
TIMI UA/NSTEMI (in-hospital)	0.27	9.93
TIMI UA/NSTEMI (6-month)	0.02	17.55
GRACE-in hospital	0.75	5.08
GRACE-6-month	0.19	11.24
TIMI UA/NSTEMI + Killip/CHF, HR, SBP (in-hospital)	0.11	12.98
TIMI UA/NSTEMI + Killip/CHF, HR, SBP (6-month)	0.58	6.59

**Figure 5:**
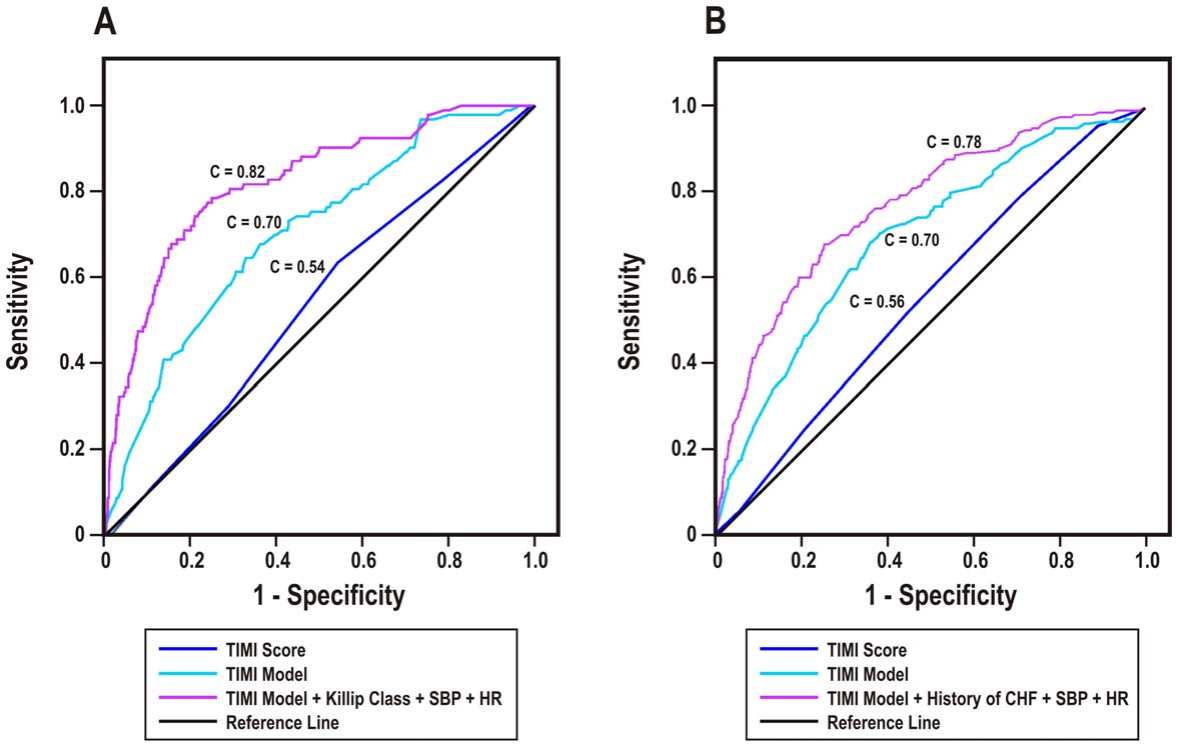
Modifications to the TIMI UA/NSTEMI risk model. Receiver operating characteristic curves of the TIMI UA/NSTEMI risk score, the TIMI UA/NSTEMI refitted multivariate model, and a modified TIMI UA/NSTEMI model including heart failure and hemodynamic variables for predicting (A) in-hospital mortality and (B) 6-month mortality in patients surviving to hospital discharge.

Independent predictors of mortality were added to the fitted, seven-variable TIMI UA/NSTEMI model to assess for improvements in model discrimination. The addition of dichotomous variables for Killip class (C = 0.78), heart rate (C = 0.73), and systolic blood pressure (C = 0.74) – each as defined for the TIMI STEMI risk score – independently enhanced model discrimination for in-hospital mortality. With all three risk factors, the revised ten-variable TIMI model was well-calibrated for in-hospital mortality (Figure S3E, Table 6) and yielded a c-statistic of 0.82 (95% CI: 0.78–0.87; Figure 5A). Although heart rate (0.71) and systolic blood pressure (0.71) added minimally to model discrimination for 6-month mortality, history of congestive heart failure (0.76) – substituted for Killip class – added noticeable discriminative power. All three risk factors contributed to a revised ten-variable TIMI model with adequate calibration (Figure S3F, Table 6) and a final c-statistic of 0.78 (95% CI: 0.75–0.81; Figure 5B).

Analysis of parsimony and fitting employing the Bayesian Information Criterion (BIC) and Akaike Information Criterion (AIC) produced similar results. Addition of the aforementioned variables to the TIMI multivariate model yielded lower BIC and AIC values (Table S2), consistent with improvements to the model and further attesting to the incremental value of these predictors.

## Discussion

Our study provides a novel comparison of both GRACE risk scores and both TIMI risk scores in a broad-spectrum of ACS patients from a single registry, and uniquely evaluates potential reasons for the underperformance of the TIMI UA/NSTEMI risk score in this study population. Specifically, we demonstrate that the GRACE risk scores are superior to the TIMI UA/NSTEMI score, but comparable to the TIMI STEMI score in the prediction of in-hospital and 6-month mortality. Moreover, the full, re-fitted TIMI UA/NSTEMI multivariate model surpasses the TIMI UA/NSTEMI risk score in discriminative capacity and improves greatly with the inclusion of heart rate, systolic blood pressure, and either Killip class (for in-hospital mortality) or history of CHF (for 6-month mortality).

### Risk score comparisons

Prior comparisons of these risk scores in NSTEMI and cardiac chest pain patients have cited the greater discriminative capacity of the GRACE scores in-hospital and at 1 year for mortality alone, a composite endpoint of death and recurrent MI, and the combined triple endpoint of death, recurrent MI, and recurrent revascularization [8], [9], [10]. Concordantly, we report significantly higher c-statistics for the GRACE in-hospital and 6-month risk scores as compared with the TIMI UA/NSTEMI score for the respective endpoints of in-hospital and 6-month all-cause mortality.

Our study represents the first direct comparison of the TIMI STEMI risk score with the two GRACE risk scores for predicting in-hospital and 6-month mortality within a STEMI subpopulation. Consistent with previous validation studies [16], [17], but in contrast to its UA/NSTEMI counterpart, the TIMI STEMI score showed excellent discrimination for in-hospital and 6-month mortality, comparable to that of the appropriate GRACE risk scores at each time point.

### Endpoint considerations

Although the TIMI UA/NSTEMI score was created for a composite endpoint of mortality, recurrent MI, and repeat revascularization, the difficulties associated with such combined endpoints – i.e. regional treatment biases including availability of catheterization laboratories – have been previously noted [7], [18]. Moreover, the TIMI UA/NSTEMI score showed better discrimination for mortality alone (C = 0.74) than for the composite endpoint (C = 0.65) within its original validation cohort [2]. Our observations are comparable to those of prior studies that have reported modest c-statistics for the TIMI UA/NSTEMI score despite using a combined endpoint of death and recurrent MI (C = 0.59 at 1-month [17], and C = 0.585 [8] or 0.62 [17] at 1-year).

Also, while the TIMI UA/NSTEMI and STEMI scores were developed for shorter endpoints – 14 days and 30 days, respectively – prior studies have assessed the TIMI UA/NSTEMI score at longer time-points, including at 3-months and at 1-year [8], [9], [10]. Since a significant risk of adverse events in ACS persists after the first 30 days, we proceeded to assess these risk scores for the 6-month endpoint.

### Model simplicity, model composition, and variable selection

A risk score's ease of use generally compromises its prognostic accuracy, as does application of a risk score to a population distinct from its derivation cohort [19], [20]. A full multivariate model is therefore more accurate than its corresponding risk score, and refitting of an externally-derived multivariate model to the study population further improves model discrimination [21], [22]. Accordingly, in our analysis, refitted multivariate models conferred a consistent discriminative improvement over their corresponding risk scores, most noticeably in the TIMI UA/NSTEMI model.

Despite its improvement from the corresponding risk score, the discriminative capacity of the refitted TIMI UA/NSTEMI multivariate model (C = 0.70 in-hospital and at 6-months) remained well below that of the GRACE in-hospital and 6-month models (C = 0.88 and 0.83, respectively), suggesting the limitations of the seven TIMI UA/NSTEMI variables within the study cohort. However, inclusion of key heart failure and hemodynamic variables markedly improved discrimination in the TIMI UA/NSTEMI model. Thus, model composition may contribute more than model simplicity to the observed discriminative deficiency of the TIMI UA/NSTEMI score in our ACS population.

In a recent study of NSTEMI patients, Khot et al. highlighted five variables that provided more than 70% of the prognostic information for 30-day and 6-month mortality: age, Killip class, heart rate, systolic blood pressure, and ST depression on electrocardiogram [11]. Of note, these five variables are represented in the TIMI STEMI, GRACE in-hospital and GRACE 6-month risk scores, all of which displayed excellent discrimination in our ACS population; the TIMI UA/NSTEMI score, however, contains just two of these five variables (age and ST deviation).

Hemodynamic variables were likely excluded from the TIMI UA/NSTEMI score to restrict the use of continuous variables and maintain risk score simplicity for routine clinical use [23]. The absence of heart failure parameters may be partly attributed to inherent differences between clinical trial and community-based ACS populations. Registry populations are generally sicker than clinical trial-derived cohorts, which typically exclude patients with comorbidities such as heart failure and renal impairment [24]. Unfortunately, baseline hemodynamic, heart failure, and renal function variables were not reported in the original TIMI 11B study to be able to compare associated presenting conditions across the four derivation cohorts (Table S1). It is likely, however, that limited enrollment of advanced heart failure patients in the TIMI 11B trial precluded the incorporation of related variables into the final, seven-variable TIMI UA/NSTEMI risk score [2], [23], [25]. Thus, a risk score derived from a relatively healthier trial population may not be applicable to patients seen in routine clinical practice.

Our analysis supports the importance of Killip class/CHF, systolic blood pressure and heart rate – the three missing risk factors from the TIMI UA/NSTEMI score – by showing their pronounced incremental value to the TIMI UA/NSTEMI multivariate model. Even dichotomous versions of these normally continuous risk factors greatly improved model discrimination (C = 0.82, in-hospital mortality; C = 0.77, 6-month mortality) while preserving the simplicity of the TIMI UA/NSTEMI score. In fact, the improvements in AIC and BIC attest to the inherent benefit of these added predictors independent of the number of variables in the revised model.

### Limitations

The present analyses derive from an observational, single institution study with a relatively small sample size and was thus subject to various unaccounted confounders inherent to such investigations. Furthermore, the endpoint differences noted previously between the TIMI risk scores and those employed in the study must be taken into consideration.

### Conclusions

In accordance with prior findings, we demonstrate the discriminative benefit of the GRACE scores over the TIMI UA/NSTEMI score for predicting mortality in our unselected, community-derived cohort of UA/NSTEMI patients. Additionally, we report similar prognostic capabilities between the GRACE scores and the TIMI STEMI score in STEMI patients. Due to their consistent discriminative accuracy in broad spectrum ACS populations, the GRACE scores may represent the most reliable risk stratification tools for ACS patient management.

Our findings also suggest that a risk score's accuracy need not be compromised significantly by its ease of use, provided it is comprised of the appropriate predictors. In fact, the sustained robustness of the TIMI STEMI score, despite its simplicity, lends considerable support to the importance of variable selection and model composition in the development of risk scores. Considering the observed benefits of heart failure and hemodynamic variables to the TIMI UA/NSTEMI score, it may be both preferable and feasible to develop a revised risk stratification tool for UA/NSTEMI patients combining the discriminative power of the GRACE scores with the inherent simplicity of the TIMI scores.

## Supporting Information

Figure S1 Plots of observed versus predicted mortality in UA/NSTEMI patients for: the GRACE risk scores at (A) in-hospital and (B) 6-month time-points; and for the TIMI UA/NSTEMI risk score at (C) in-hospital and (D) 6-month time-points.
(0.09 MB DOC)

Figure S2 Plots of observed versus predicted mortality in STEMI patients for: the GRACE risk scores at (A) in-hospital and (B) 6-month time-points; and for the TIMI STEMI risk score at (C) in-hospital and (D) 6-month time-points.
(0.08 MB DOC)

Figure S3 Plots of observed versus predicted mortality in UA/NSTEMI patients for: the TIMI UA/NSTEMI refitted multivariate model at (A) in-hospital and (B) 6-month time-points; the GRACE refitted multivariate models at (C) in-hospital and (D) 6-month time-points; and the revised, 10-variable TIMI UA/NSTEMI refitted multivariate model at (E) in-hospital and (F) 6-month time-points.
(0.10 MB DOC)

Figure S4 Receiver operating characteristic curves of (A) the TIMI UA/NSTEMI and GRACE in-hospital refitted multivariate models for predicting in-hospital mortality, and (B) the TIMI UA/NSTEMI and GRACE 6-month refitted multivariate models for predicting 6-month mortality in patients surviving to hospital discharge.
(2.18 MB TIF)

Table S1 Baseline characteristics of patients in risk score derivation cohorts.
(0.05 MB DOC)

Table S2 AIC/BIC values for improvements to TIMI model.
(0.04 MB DOC)
