# Supplementary material for: Does Simplicity Compromise Accuracy in ACS Risk Prediction? A Retrospective Analysis of the TIMI and GRACE Risk Scores
Source: PLoS One. 2009 Nov 23;4(11):e7947. doi: 10.1371/journal.pone.0007947 (PMC2776353; doi:10.1371/journal.pone.0007947)

**Figure S2 – STEMI risk score calibration plots**

**STEMI**

**GRACE** – (A) in-hospital and (B) 6-month mortality

1. (B)


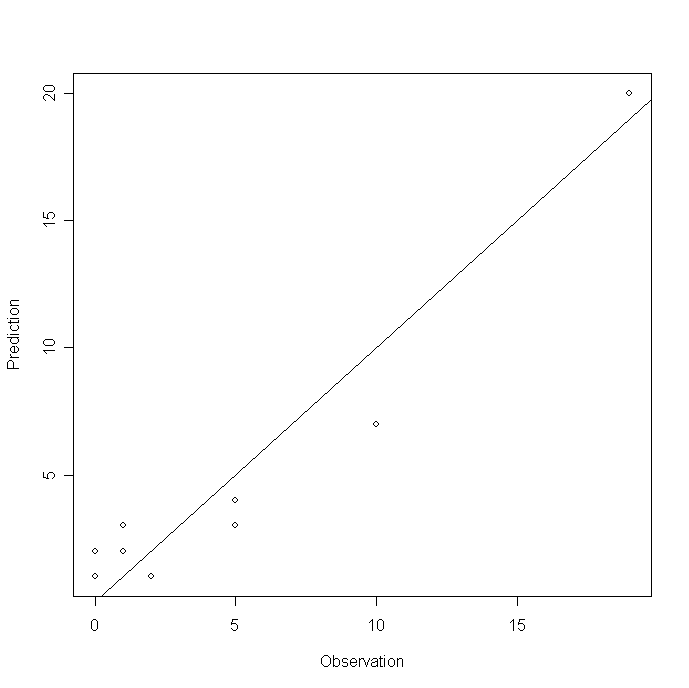

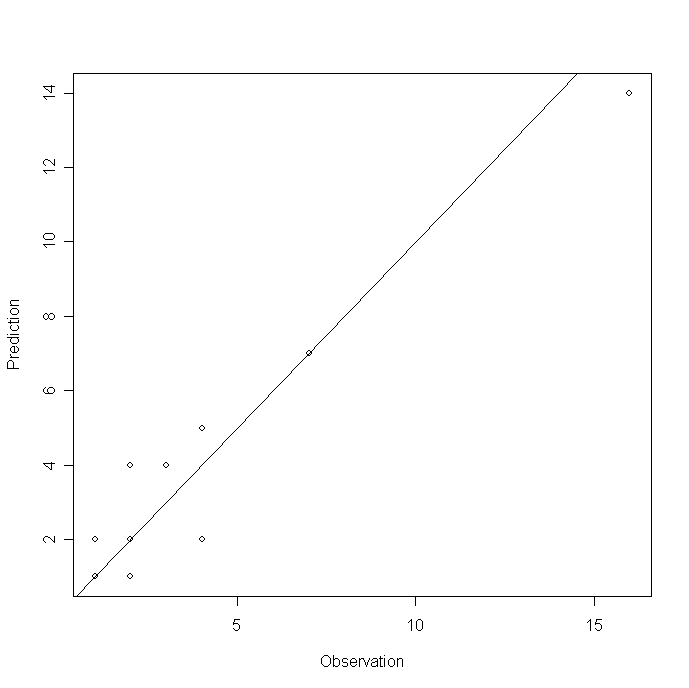


**TIMI STEMI** – (C) in-hospital and (D) 6-month mortality

(C) (D)


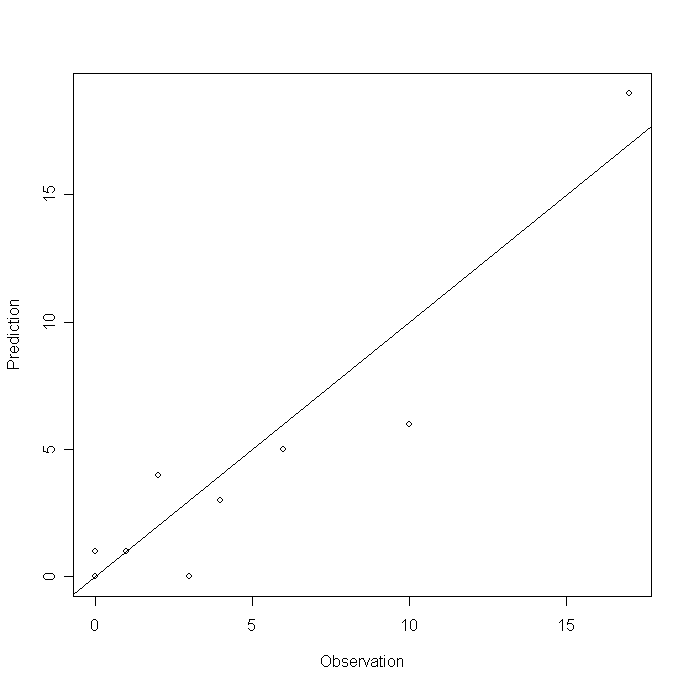

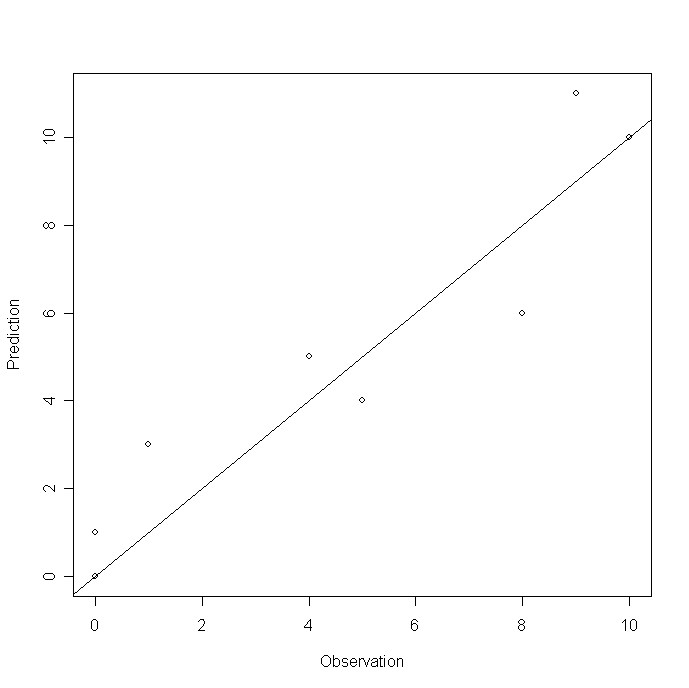

Supplement: Figure S2 — Plots of observed versus predicted mortality in STEMI patients for: the GRACE risk scores at (A) in-hospital and (B) 6-month time-points; and for the TIMI STEMI risk score at (C) in-hospital and (D) 6-month time-points. (0.08 MB DOC) [file pone.0007947.s002.doc]
