# Supplementary material for: Does Simplicity Compromise Accuracy in ACS Risk Prediction? A Retrospective Analysis of the TIMI and GRACE Risk Scores
Source: PLoS One. 2009 Nov 23;4(11):e7947. doi: 10.1371/journal.pone.0007947 (PMC2776353; doi:10.1371/journal.pone.0007947)

**Figure S3 - UA/NSTEMI refitted multivariate model calibration plots**

**TIMI UA/NSTEMI** – (A) in-hospital and (B) 6-month mortality

1. (B)


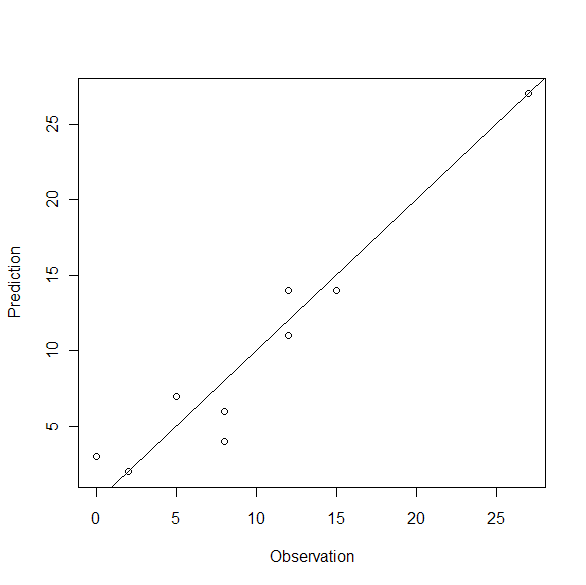

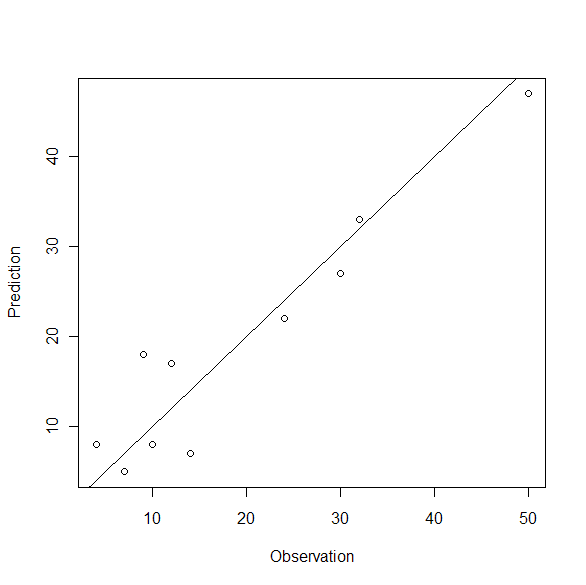


**GRACE** – (C) in-hospital and (D) 6-month mortality

(C) (D)


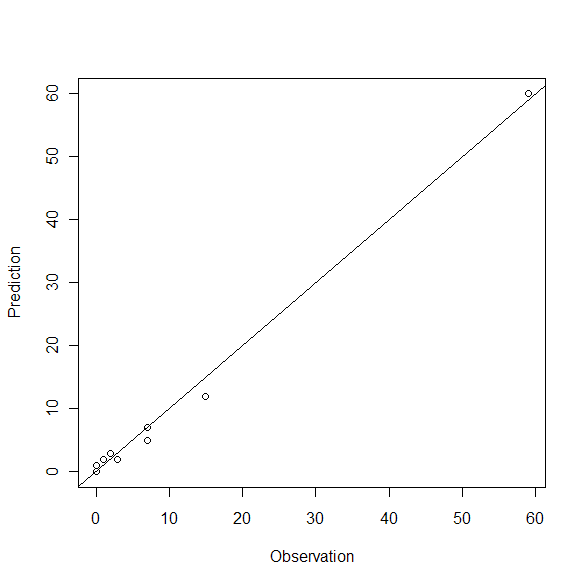

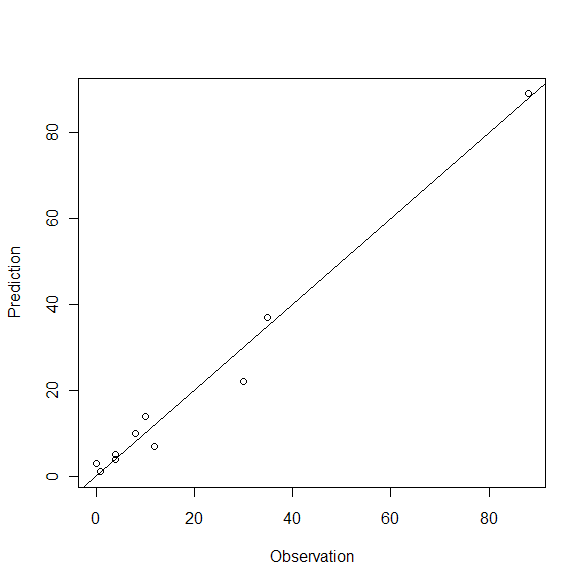


**TIMI UA/NSTEMI plus Killip/CHF, HR, and SBP** – (E) in-hospital and (F) 6-month mortality

(E) (F)


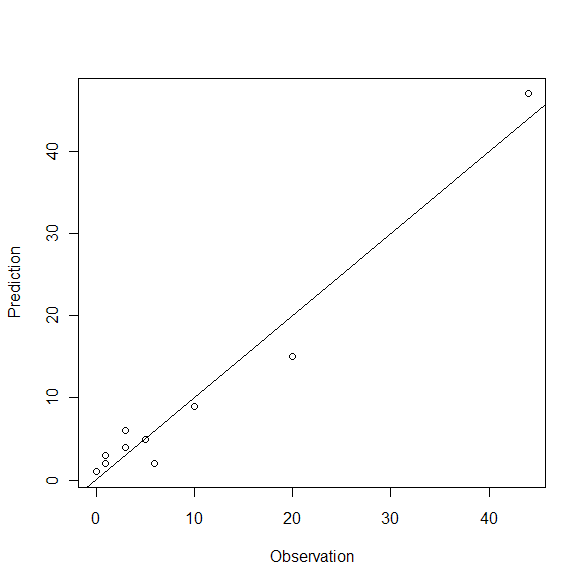

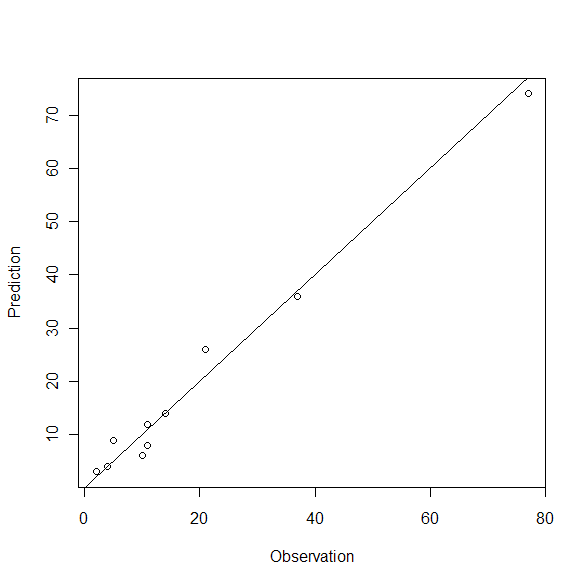

Supplement: Figure S3 — Plots of observed versus predicted mortality in UA/NSTEMI patients for: the TIMI UA/NSTEMI refitted multivariate model at (A) in-hospital and (B) 6-month time-points; the GRACE refitted multivariate models at (C) in-hospital and (D) 6-month time-points; and the revised, 10-variable TIMI UA/NSTEMI refitted multivariate model at (E) in-hospital and (F) 6-month time-points. (0.10 MB DOC) [file pone.0007947.s003.doc]
