# Supplementary figures and images for: Does Simplicity Compromise Accuracy in ACS Risk Prediction? A Retrospective Analysis of the TIMI and GRACE Risk Scores
Source: PLoS One. 2009 Nov 23;4(11):e7947. doi: 10.1371/journal.pone.0007947 (PMC2776353; doi:10.1371/journal.pone.0007947)

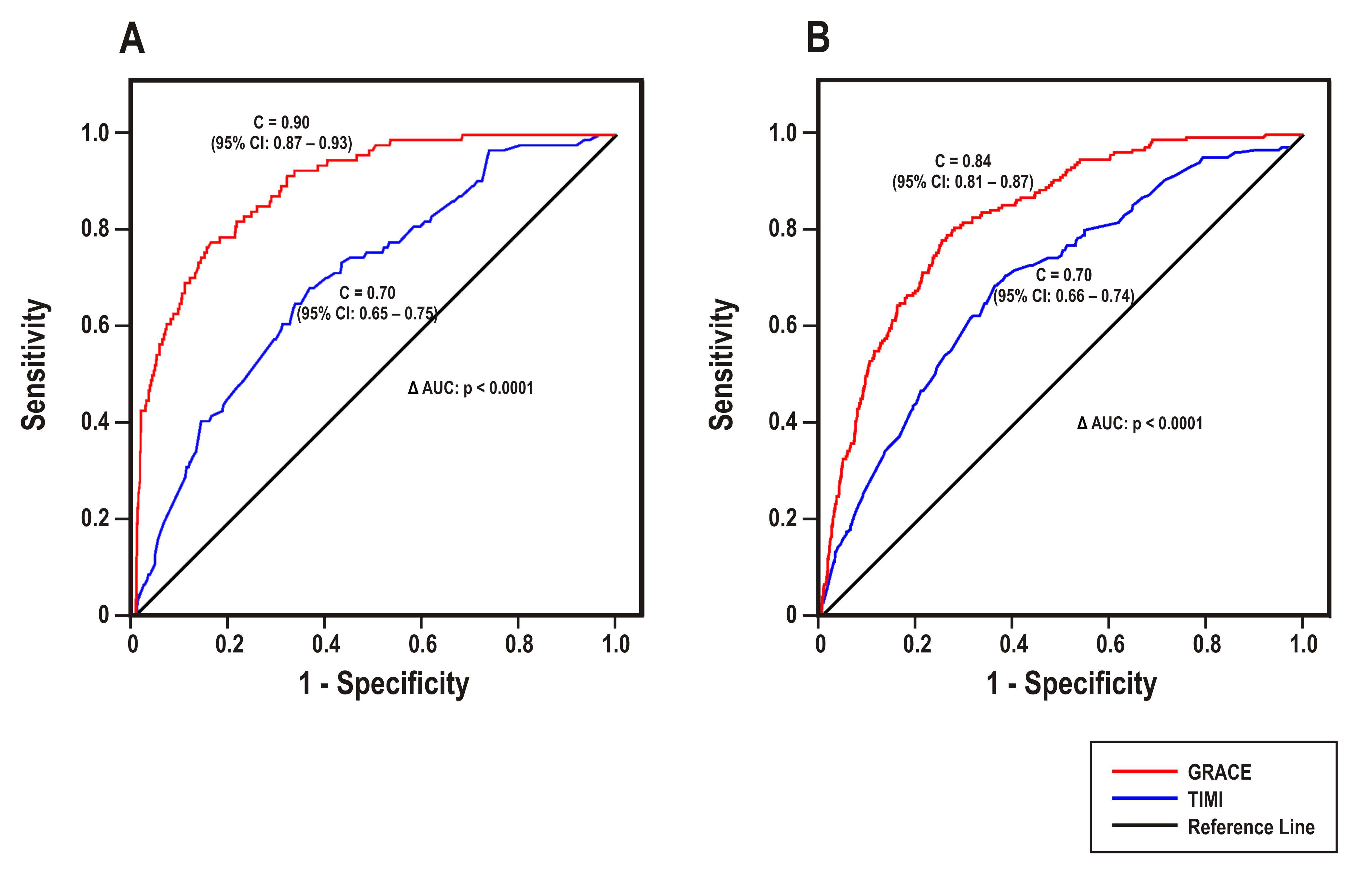

Supplement: Figure S4 — Receiver operating characteristic curves of (A) the TIMI UA/NSTEMI and GRACE in-hospital refitted multivariate models for predicting in-hospital mortality, and (B) the TIMI UA/NSTEMI and GRACE 6-month refitted multivariate models for predicting 6-month mortality in patients surviving to hospital discharge. (2.18 MB TIF) [file pone.0007947.s004.tif]
