# Supplementary material for: Does Simplicity Compromise Accuracy in ACS Risk Prediction? A Retrospective Analysis of the TIMI and GRACE Risk Scores
Source: PLoS One. 2009 Nov 23;4(11):e7947. doi: 10.1371/journal.pone.0007947 (PMC2776353; doi:10.1371/journal.pone.0007947)
Supplement: Table S1 — Baseline characteristics of patients in risk score derivation cohorts. (0.05 MB DOC) [file pone.0007947.s005.doc]

**Table S1 – Baseline characteristics of patients in risk score derivation cohorts**

| **Clinical characteristics** | **TIMI 11B – UFH group**  (TIMI UA / NSTEMI score derivation cohort)  **n = 1957** | **In-TIME-II**  (TIMI STEMI score derivation cohort)  **n = 15060** | **GRACE Registry**  (GRACE in-hospital score derivation cohort)  **n = 11389** | **GRACE Registry (**GRACE 6-month score derivation cohort)  **n = 15007** |
| --- | --- | --- | --- | --- |
| Age | 66 (57, 72) | 62 (52, 70) | 66.3 (56, 75) | 66 (55.5, 74.6) |
| Female | 35.8 | 24.7 | 33.5 | 33.2 |
| Weight | 77 (68, 87) | 77 (69,86) | 76 (67, 86) | - - |
| **Medical History** |  |  |  |  |
| Hypertension | 49.8 | 30.4 | 57.8 | 58.2 |
| Hyperlipidemia | 31.6 | - - | 43.6 | 45.6 |
| Diabetes Mellitus | 20.1 | 13.9 | 23.3 | 23.5 |
| Myocardial infarction (MI) | 32.3 | 16.0 | 32.0 | 32.0 |
| Percutaneous coronary intervention (PCI) | 11.9 | 4.5 | 14.0 | 15.3 |
| Coronary artery bypass graft (CABG) | 13.2 | 2.7 | 12.6 | 13.4 |
| **Presentation** |  |  |  |  |
| Initial cardiac markers | 39.6 | - - | 31.6 | 33.6 |
| ST-deviation | 72.3 | - - | 54.1 | 52.5 |
| ST-depression | 55.2 | - - | 33.7 | 32.1 |
| Killip class II-IV | - - | 12.6 | 17.3 | 15.8 |
| Heart rate | - - | 74 (63, 86) | 76 (65, 90) | 76 (65, 89) |
| Systolic BP | - - | 140 (122, 155) | 140 (120, 160) | 140 (122, 160) |
| **Prior Medical Therapy** |  |  |  |  |
| Aspirin | - - | - - | 43.0 | 43.4 |
| Beta-blocker | - - | 15.6 | - - | 30.8 |
| ACE inhibitors | - - | - - | 26.0 | 25.6 |
| Statins | - - | 9.3 | 20.4 | 22.6 |

Continuous variables reported as: Median (interquartile range)

Categorical variables reported as: % of population
