# Supplementary material for: Does Simplicity Compromise Accuracy in ACS Risk Prediction? A Retrospective Analysis of the TIMI and GRACE Risk Scores
Source: PLoS One. 2009 Nov 23;4(11):e7947. doi: 10.1371/journal.pone.0007947 (PMC2776353; doi:10.1371/journal.pone.0007947)
Supplement: Table S2 — AIC/BIC values for improvements to TIMI model. (0.04 MB DOC) [file pone.0007947.s006.doc]

**Table S2 – AIC/BIC values for improvements to TIMI model**

**(A)**

| **In-hospital Models** | **C-statistic** | **AIC** | **BIC** |
| --- | --- | --- | --- |
| 7 TIMI UA/NSTEMI variables | 0.70 | 786.40 | 833.76 |
| 7 TIMI UA/NSTEMI variables + **Killip class** | 0.78 | 725.13 | 778.29 |
| 7 TIMI UA/NSTEMI variables + **Heart Rate** | 0.74 | 768.62 | 821.91 |
| 7 TIMI UA/NSTEMI variables + **Systolic Blood Pressure** | 0.74 | 749.92 | 803.21 |
| 7 TIMI UA/NSTEMI variables + **Killip, HR and SBP** | 0.82 | 683.13 | 748.10 |

(B)

| **Six-month follow-up Models** | **C-statistic** | **AIC** | **BIC** |
| --- | --- | --- | --- |
| 7 TIMI UA/NSTEMI variables | 0.70 | 1289.33 | 1336.07 |
| 7 TIMI UA/NSTEMI variables + **Heart Rate** | 0.71 | 1276.36 | 1328.93 |
| 7 TIMI UA/NSTEMI variables + **Systolic Blood Pressure** | 0.71 | 1276.96 | 1329.54 |
| 7 TIMI UA/NSTEMI variables + **History of CHF** | 0.77 | 1194.84 | 1247.42 |
| 7 TIMI UA/NSTEMI variables + **History of CHF, HR and SBP** | 0.78 | 1178.90 | 1243.16 |
